# Molecular Basis of Water Activity in Glycerol–Water Mixtures

**DOI:** 10.3389/fchem.2019.00731

**Published:** 2019-11-01

**Authors:** Hiroshi Nakagawa, Taiji Oyama

**Affiliations:** ^1^Hierarchical Structure Research Group, Materials Science Research Center, Japan Atomic Energy Agency, Ibaraki, Japan; ^2^JASCO Corporation, Tokyo, Japan

**Keywords:** water activity, isotherm sorption, water dynamics, glycerol–water mixture, DSC, ATR-IR, IQENS

## Abstract

Water activity (Aw) is a reliable indication of the microbial growth, enzymatic activity, preservation, and quality of foods. However, a molecular basis of Aw is still under debate in multiple related disciplines. Glycerol–water mixtures can provide a variation of Aws by controlling the ratio of glycerol and water. In this study, the molecular basis of Aw was examined by using differential scanning calorimetry (DSC), attenuated total reflection Fourier-transform infrared spectroscopy (ATR-IR), and incoherent quasi-elastic neutron scattering (IQENS) based on moisture sorption isotherms of glycerol–water mixtures. Three regions were identified and classified based on DSC results. DSC showed that bulk-like water existed at Aw > ≈ 0.7 at 27°C. Hydrogen bonding related molecular vibrations were analyzed by ATR-IR, which indicated that the OH stretching in water molecules is significantly different for Aw > ≈ 0.7. Translational diffusive and/or rotational motions in time and space analyzed by IQENS appeared when Aw > ≈ 0.7, and are correlated with hydrogen bonding related local vibrational dynamics in the glycerol–water mixtures. More importantly, Aw values of glycerol–water mixtures can be explained by the hydrogen bonding network and molecular dynamics of water in the solution. We discuss the implications of Aw in the preservation of food at the molecular level.

## Introduction

The interactions between water molecules and biomaterials and ingredients in food are crucial in life and food sciences. The concept of water activity (Aw) was introduced over 60 year ago (Scott, 1953) and has been used as a practical and reliable indication of the microbial growth, enzymatic activity, preservation, and quality of food (Roos, 1993; Sablani et al., 2007). The Aw is a thermodynamic value and is defined as the ratio of the equilibrated vapor pressure (*P*_1_) of the sample to the saturation of vapor pressure (*P*_0_) of pure water at the same temperature, Aw = *P*_1_/*P*_0_. It has been widely used in the food industry as well as basic food science. It has been demonstrated that solute–solute and solute–water interactions contribute to the Aw (Maneffa et al., 2017). However, limitations of the Aw concept have also been discussed (Rahman, 2009). At the molecular scale, a free volume model was applied to interpret and predict Aw (He et al., 2006). The molecular background of Aw is an essential topic (Renshaw et al., 2019). Yet, the molecular basis of Aw is still under debate in multiple related disciplines.

Glycerol–water mixtures are used to control the equilibrium relative humidity (RH) (Forney and Brandl, 1992; Marcolli and Peter, 2005). In the equilibrated state, RH = Aw. The Aw of the solutions is changed from 0.0 to 1.0, depending on the glycerol fraction. Glycerol and water can be easily mixed and is inexpensive. Due to the advantageous sample preparation, this system has been well studied experimentally and theoretically from various viewpoints such as the molecular structure, dynamics, and hydrogen bond network (Hayashi et al., 2005, 2006; Puzenko et al., 2005, 2007; Dashnau et al., 2006; Kataoka et al., 2011; Towey et al., 2011a,b; Murata and Tanaka, 2012; Towey and Dougan, 2012). In most research, however, the physical and chemical properties were examined as a function of molar fraction of glycerol, and there are few arguments regarding the relationship with Aw. Glycerol is a typical compatible solute in a biological system (Dashnau et al., 2006). In order to clarify the physiological roles of glycerol, it is essential to understand the interaction between water and glycerol from the dynamical point of view. Differential scanning calorimetry (DSC) measurements of water–glycerol mixtures showed that water in higher glycerol concentration solutions goes into a super-cooling state, and it was discussed that this physical property of water is likely related to the hydrogen-bonding network of water molecules and its interaction with solutes (Hayashi et al., 2006). The physical properties of water in the glycerol–water mixtures are biologically and chemically attractive (Morris et al., 2006; Furushima et al., 2012; Tanaka et al., 2013, 2015; Prickett et al., 2015; Bag and Valenzuela, 2017; Jang et al., 2017; Khan and Tanaka, 2018).

The properties of the glycerol–water mixtures have been examined by a number of methods including attenuated total reflection Fourier-transform infrared spectroscopy (ATR-IR) (Zelent et al., 2004; Kitadai et al., 2014), Nuclear Magnetic Resonance (NMR) (D'Errico et al., 2004), broadband dielectric spectroscopy (Behrends et al., 2006; Hayashi et al., 2006), electron spin resonance (Banerjee and Bhat, 2009), neutron diffraction (Towey et al., 2011a,b) thermodynamic analysis (Popov et al., 2015), and molecular dynamics simulation (Dashnau et al., 2006; Dashnau and Vanderkooi, 2007; Egorov et al., 2011). Incoherent quasi-elastic neutron scattering (IQENS) should be compatible to these methods (Jansson and Swenson, 2008) and is promising method for studying the molecular dynamics of the glycerol–water mixtures. These methods can be used to analyze how water and its properties interact with biomaterials and foods. IQENS probes the self-correlation of a single particle, which is useful for characterizing molecule dynamics. The hydrogen atom has a large cross section in IQENS. Furthermore, the cross section of hydrogen is larger than that of deuterium by one order of magnitude. Therefore, the molecular dynamics of a specific molecule in a multiple component system can be separately observed by deuteration isotope labeling (Nakagawa and Kataoka, 2010, in press).

In this study, we examined the thermodynamics and molecular dynamics of glycerol–water mixtures as a function of Aw, and discussed the molecular basis of Aw. This is thermodynamic parameter, which should be more directly relevant to the molecular dynamics rather than the molecular structure from the view point of statistical mechanics. The states of water in the mixtures were classified into three regions in the isotherm sorption curve. The thermodynamics were examined by DSC, and local dynamics of the mixtures, such as the hydrogen bonding of water and skeletal fluctuation of glycerol, were examined by ATR-IR. The translational and/or rotational motions were analyzed by IQENS. It was found that the obtained results were correlated, which suggests that thermodynamics and molecular dynamics should be connected in the glycerol–water mixture system. This discussion provides important insight for the molecular basis of Aw. Finally, the implications of Aw in the preservation of food are discussed from the molecular viewpoint.

## Materials and Methods

### Materials

Glycerol purchased from Wako Pure Chemical Industries Ltd. was used without further purification. The glycerol–water mixtures with glycerol content between 0 and 100 vol.% in 5 vol.% intervals were prepared with double distilled water. The corresponding mol.% of each sample is listed in Table 1, which can be used to compare the present data with that in the literature. No contamination of water in the glycerol was confirmed by checking the H_2_O signal in FT-IR spectra.

**Table 1:** Mole fraction of glycerol (x_gly_), volume fraction (v_gly_) of glycerol, and water activities (Aw) of the glycerol–water mixtures at 25°C.

**v_**gly**_**	**x_**gly**_**	**Aw**	**v_**gly**_**	**x_**gly**_**	**Aw**
1.00	1.00	0.05	0.45	0.17	0.80
0.95	0.83	0.14	0.40	0.14	0.83
0.90	0.69	0.25	0.35	0.12	0.86
0.85	0.58	0.34	0.30	0.10	0.88
0.80	0.50	0.42	0.25	0.08	0.91
0.75	0.43	0.50	0.20	0.06	0.93
0.70	0.37	0.56	0.15	0.04	0.95
0.65	0.32	0.62	0.10	0.03	0.96
0.60	0.27	0.68	0.05	0.01	0.97
0.55	0.23	0.73	0.00	0.00	0.98
0.50	0.19	0.77			

### Water Activity (Aw)

The water activity values, Aw, were measured at 27°C using water activity equipment (LabSwift-aw, Novasina, Switzerland). The instrument was calibrated by the instrument company, and the instrumental condition was checked by the standard capsules with some water activities just before the sample measurement. The measurements were repeated three times for each sample, and the uncertainty was about 0.002. The Aw for each glycerol mol.% is listed in Table 1.

### Differential Scanning Calorimetry (DSC)

DSC measurements were performed by a Thermo plus EVO2 DSC8231LN (Rigaku) from −130°C to 60°C with a cooling and heating rate of 5°C/min. An empty aluminum pans was used as a reference.

### Attenuated Total Reflection Fourier-Transform Infrared Spectroscopy (ATR-IR)

ATR-IR measurements were performed using an FTIR spectrometer (FTIR-6600; Jasco Corp.). Diamond attached to an ATR plate with a horizontal ATR accessory was used for measurements. All spectra were obtained with a spectra range of 400–7,800 cm^−1^ with 4 cm^−1^ resolution. The background spectra were measured on the ATR plate without a sample every two sample measurements. The spectra were taken at 27°C (room temperature) and under nitrogen flow to minimize the effects of water vapor and CO_2_. Sample spectra were divided by the background spectrum, and then the obtained spectra were treated by ATR-correction. The ATR-IR spectra were recorded as absorbance.

### Incoherent Quasi-Elastic Neutron Scattering (IQENS) Experiment

Neutron scattering experiments were performed with the triple axis spectrometer LTAS in the JRR-3M reactor, which has an energy resolution of ca. 106 μeV, in Tokai, Japan. The energy resolution was determined by measuring a vanadium. The measured *Q*-value was fixed to 1.7 Å^−1^ in order to detect both the translational and/or rotational motions effectively, because the corresponding correlation length, 3.6 Å, is roughly comparable to the size of a few water molecules. Neutron scattering measurements were performed at 27°C. The obtained data were corrected by empty cell background subtraction. Data analysis was performed without multiple scattering correction because the sample transmission value was relatively large, approximately 90%. The measured samples were (glycerol-h8 + H_2_O), (glycerol-d8 + H_2_O), and (glycerol-d8 + D_2_O) over a series of glycerol concentrations. The dynamics of the glycerol–water mixtures were analyzed with (glycerol-h8 + H_2_O) samples (representing contribution from both glycerol and water). In order to selectively observe the dynamics of water alone in the mixtures, the neutron spectra were obtained by the difference in INS between (glycerol-d8 + H_2_O) and (glycerol-d8 + D_2_O), as follows (Nakagawa and Kataoka, 2010, in press),

(1) $$\begin{array}{l} S{\left(Q,\omega \right)}_{water}=S{\left(Q,\omega \right)}_{\left(glycerol-d8\;+\;H2O\right)}\\ \;-S{\left(Q,\omega \right)}_{\left(glycerol-d8\;+\;D2O\right)}. \end{array}$$

## Results and Discussion

### Sorption Isotherm

**Figure 1:**
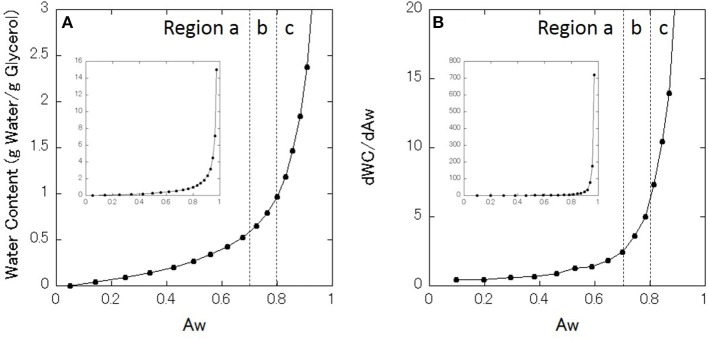
**(A)** Moisture sorption isotherm of glycerol–water mixtures. **(B)** First derivative of the sorption isotherm. Regions a, b, and c are classified. Inset indicate the all data in the full range.

Table 1 lists the measured values of Aw for each glycerol–water mixture at 27°C. The Aw of the mixtures at 25°C are presented in the literature (Marcolli and Peter, 2005), and the present results are almost consistent with the data in the literature. The data in Table 1 is presented in Figure 1A as the sorption isotherm. This figure shows that the water content monotonously increases with increasing Aw, and water content increases more steeply for higher Aw. Figure 1B shows the first derivative of the sorption isotherm curve. For convenience, two boundary values of Aw are determined based on the DSC data at around 0.7 and 0.8 (see DSC results below), around which three regions are defined as region a, b, and c.

### Glass Transition, Melting, and Freezing Temperatures

**Figure 2:**
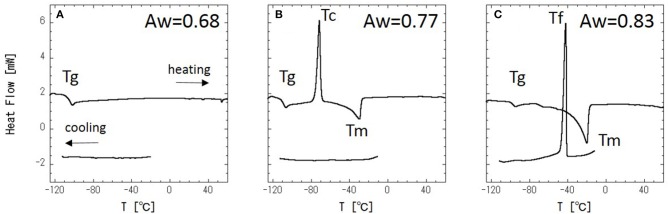
DSC curves of glycerol–water mixtures at Aw = 0.68 **(A)**, 0.77 **(B)**, and 0.83 **(C)**.

**Figure 3:**
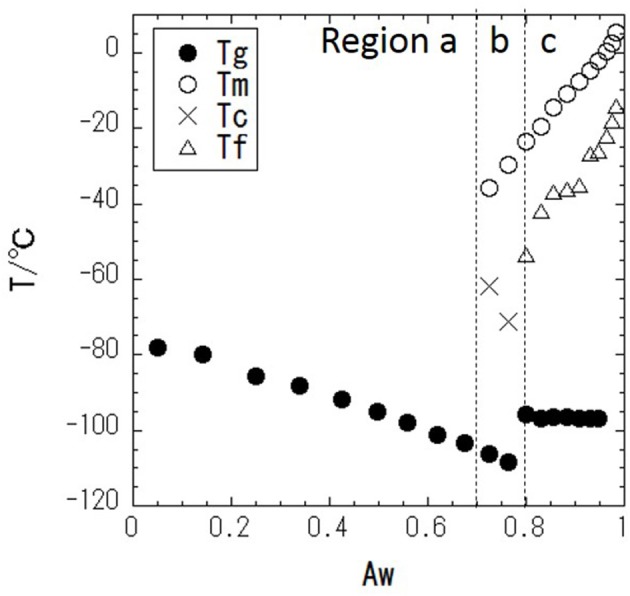
Phase diagram of glycerol–water mixture with temperature (T/°C) vs. water activity (Aw); • is the glass transition temperature, Tg, ○ is the melting temperature, Tm, × is the cold-crystallization temperature, Tc, and Δ is the freezing temperature, Tf.

DSC measurements were performed with a series of glycerol–water mixtures. Typical DSC data are shown in Figure 2. When Aw = 0.68 (Figure 2A), there is no DSC signal during cooling, and a glass transition is observed during heating. This result indicates that water in the mixture is super-cooled upon cooling. Figure 2B shows the DSC results at Aw = 0.77, where there is, again, no DSC signal during cooling, while a cold-crystallization peak corresponding to super-cooled water is observed at −71°C during heating. The cold-crystallization is also observed at Aw = 0.73, but this phenomena is observed within narrow Aw range. The intermediate region, where the cold-crystallization is observed, should correspond to the regions where liquid-liquid transition (LLT) exists (Murata and Tanaka, 2012; Popov et al., 2015). It has also been discussed that this phenomenon should indicate an intermediate water phase exists (Tanaka et al., 2013, 2015; Bag and Valenzuela, 2017). For Aw = 0.83 (Figure 2C), ice crystallization is observed during cooling, and then a glass transition and melting of the ice are observed during heating. The glass transition of glycerol (Tg), melting of ice (Tm), freezing of water (Tf), and cold-crystallization of water (Tc) are shown in Figure 3.

### ATR-IR

**Figure 4:**
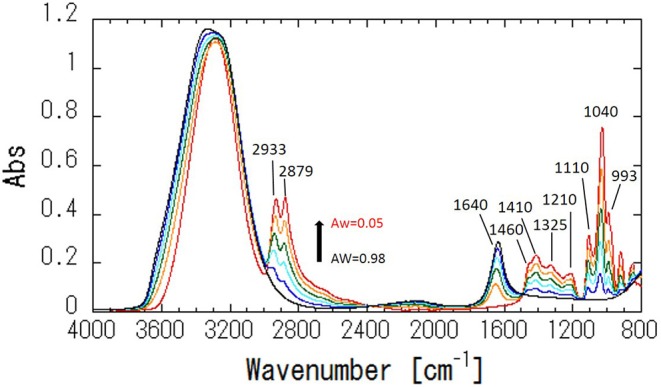
ATR-IR spectra of glycerol–water mixtures at Aw = 0.05, 0.42, 0.68, 0.83, 0.93, and 0.98.

**Figure 5:**
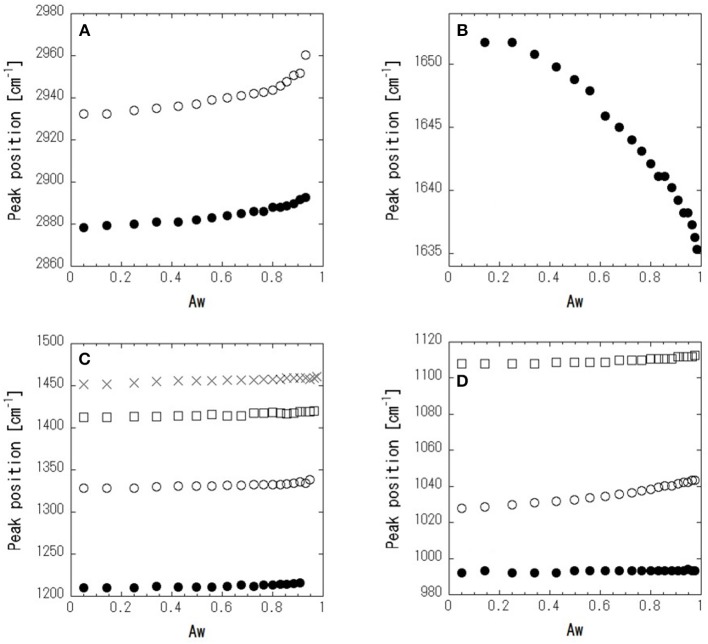
Peak positions as a function of Aw for symmetric (•) and asymmetric (○) CH_2_-stretching **(A)**, HOH-bending **(B)**, bending of CH_2_, (× and □) in plane rocking of OH (○), and stretching of CCC and COC (•) **(C)**, and stretching of C–O in CHOH (□) and in CH_2_OH (○ and •) **(D)**.

**Figure 6:**
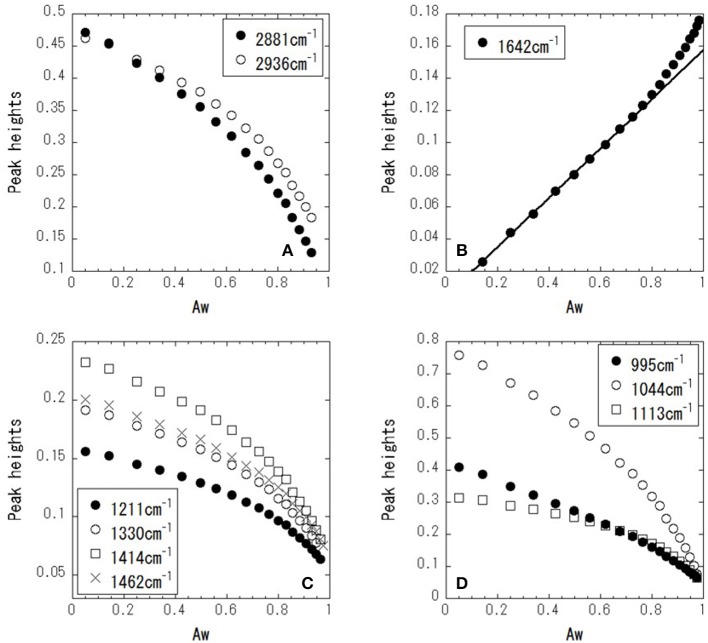
Peak heights as a function of Aw for 2,881 and 2,936 cm^−1^ **(A)**, 1,642 cm^−1^ **(B)**, 1,221, 1,330, 1,414, and 1,462 cm^−1^ **(C)**, and 995, 1,044, and 1,113 cm^−1^ **(D)**.

Figure 4 shows ATR-corrected spectra of mixtures covering a range of Aw. For Aw = 0.05, asymmetric and symmetric stretching of CH_2_ are observed at 2,933 and 2,879 cm^−1^, respectively. The peaks observed between 950 and 1,500 cm^−1^ are assigned to the molecular motion of glycerol. The assignments are listed in Table 2. In glycerol–water mixtures and pure water, the HOH-bending peak is observed at approximately 1,640 cm^−1^. Figure 5 shows the peak positions as a function of Aw. The peak positions of asymmetric and symmetric stretching of CH_2_ shifted to higher wavenumber with increasing Aw (Figure 5A). This shift is likely attributed to formation of hydrogen bonding between the glycerol CH_2_ groups and OH groups of water (Dashnau et al., 2006; Kataoka et al., 2011). The peak position of HOH-bending shifts to lower wavenumber for higher Aw (Figure 5B). This indicates weaker hydrogen bonding at higher Aw (Dashnau et al., 2006; Kataoka et al., 2011). In the regions b and c, the shift to lower wavenumber is more significant compared with that in region a. This is indicative of weaker hydrogen bonding between water and glycerol or between water molecules (Kataoka et al., 2011). Figures 5C,D shows that the characteristic frequency is almost independent of Aw, which suggest the relevant local dynamics should be decoupled with water dynamics. Figure 6 show the peak intensities as a function of Aw. The peak heights assigned to the molecular motion of glycerol decrease for higher Aw (Figures 6A,C,D), while the peak heights assigned to HOH-bending mode of water increases as a function of Aw (Figure 6B). These results are consistent with the literature (Kataoka et al., 2011). It should be noted that the peak height of the HOH-bending mode shows that the deviation from the trend in region a was observed in regions b and c. This might be due to the percolation of the hydrogen bonds in water (Dashnau et al., 2006; Dashnau and Vanderkooi, 2007).

**Table 2:** Assignment of the infrared spectra of the glycerol–water mixture.

**Frequency (cm^**−1**^)**	**Assignment**
2,933	Asymmetric stretching of CH_2_
2,879	Symmetric stretching of CH_2_
1,640	Bending of HOH
1,460	Bending of CH_2_
1,410	Bending of CH_2_
1,325	In-plane rocking of OH
1,210	Stretching of CCC, COC
1,110	Stretching of C-O in CHOH
1,040	Stretching of C-O in CH_2_OH
993	Stretching of C-O in CH_2_OH

### QENS

**Figure 7:**
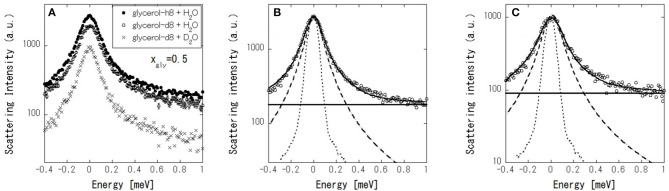
**(A)** IQENS profiles at *Q* = 1.7 Å^−1^ of (glycerol-h8 + H_2_O) (•), (glycerol-d8 + H_2_O) (○), and (glycerol-d8 + D_2_O) (×) at 0.5 mole fraction of glycerol (x_gly_). IQENS of (glycerol-h8 + H_2_O) **(B)** and (glycerol-d8 + H_2_O) – (glycerol-d8 + D_2_O) **(C)**, where open circles, dot curves, thick curves, dashed curves, and thin lines indicate the experimental data, instrumental resolution functions, curves fitted using Equation (2), Lorentzian functions in the fitting, and constant background in the fitting, respectively.

**Figure 8:**
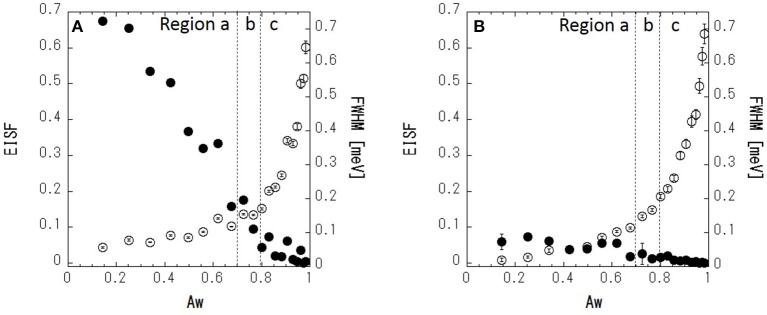
EISF and FWHM at *Q* = 1.7 Å^−1^ of (glycerol-h8 + H_2_O) **(A)** and (glycerol-d8 + H_2_O) – (glycerol-d8 + D_2_O) **(B)**. Closed (•) and open (○) circles indicate the EISF and FWHM, respectively.

Figure 7 shows the IQENS profiles of (glycerol-h8 + H_2_O), (glycerol-d8 + H_2_O), and (glycerol-d8 + D_2_O) at Aw = 0.77, where glycerol and water have equivalent molar fractions (x_gly_ = 0.5). At first, the dynamics of the glycerol–water mixtures were analyzed with (glycerol-h8 + H_2_O) samples by using the following scattering function,

(2) $$\begin{array}{l} S\left(Q,\omega \right)\;=\;A\left(Q\right)[EISF\cdot \delta \left(\omega \right)+\\ \;\left(1-EISF\right)\cdot L\left(Q,\omega \right)]⊗R\left(\omega \right)+B(\omega ), \end{array}$$

where *R*(ω) is the resolution function, ⊗ denotes the convolution, *A*(*Q*) is the Debye–Waller factor, EISF is an elastic incoherent structural factor, *L*(*Q*, ω) is a Lorentzian function with full-width at half-maximum (FWHM), and *B*(ω) is the constant background due to inelastic scattering. The scattering profile can be fitted reasonably well by Equation (2) (Figure 7B). Figure 8A shows the fitted results of EISF and FWHM. EISF decreases with increasing Aw, and in region c, EISF values are below 0.1. Glycerol, being about five times heavier than water can be expected to be slower. With a resolution of 106 μeV, which corresponds to about 6 ps, water is mobile but glycerol would be stationary on the time scales accessible to the instrument, and then glycerol dynamics can well go undetected. This should be reflected in larger EISF at lower Aw. Consequently, as the glycerol (water) content decreases (increases) at higher Aw values, the elastic contribution (and hence EISF) goes down while the quasielastic broadening (FWHM) increases. A smaller EISF means that the glycerol and water have the characteristic times of the dynamics faster than the instrumental resolution, and are moving beyond the correlation length of 3.6 (corresponding to Q = 1.7 Å^−1^ in inverse space). The FWHM increases slightly with increasing Aw in regions a and b, and increases more significantly in region c. The larger FWHM means that the characteristic time is faster for higher Aw.

Next, in order to mask the dynamics of glycerol, IQENS spectra obtained by the difference between (glycerol-d8 + H_2_O) and (glycerol-d8 + D_2_O) were analyzed using Equation (2). The obtained neutron spectra from water can be considered. The scattering profile can be fitted reasonably well by Equation (2) (Figure 7C). The fit results are shown in Figure 8B. Compared with Figure 8A, the EISF is below 0.1 over the range of Aws. This result indicates that water molecules are not confined in a space of 3.6 Å in length and might have the motion faster than the instrumental resolution, because EISF is related to the probability of finding an atom at the same position at *t* = 0 and ∞ within the dynamics window of the instrument. The FWHM in Figure 8B follows a similar trend to that in Figure 8A, showing that translational and/or rotational dynamics of water is faster at larger Aw. Q-dependence of the spectrum is essentially important to analyze the dynamics in detail, which should be future work with TOF spectrometer.

### Molecular Scale Interpretation of Water Activity in Glycerol–Water Mixtures

In this section, the physical properties of the classified water in the sorption isotherm are discussed. In region a (Aw < ≈ 0.7), there are no freezing and melting peaks in the DSC curve. This indicates that water is hydrated on glycerol, and water molecules are isolated or are present as small water clusters in the solutions, which is similar to a previous study that found small water pools isolated in the solution (Hayashi et al., 2006). Consequently, the hydrogen bonding in water with glycerol is strong. The FWHM results in the IQENS analysis show that the diffusive motion of water is slowed down by the interaction with glycerol. The strong interaction of water with glycerol leads to lower Aw. In region b (≈0.7 < Aw < ≈0.8), freezing of water (cold-crystallization) is observed in the DSC curve, suggesting that there bulk-like water is present above the melting temperature, which is in agreement with previous work in which water cooperative domains were found and water molecules percolated two-dimensionally (Hayashi et al., 2006). The cold-crystallization should be related to the liquid-liquid transition (LLT) (Murata and Tanaka, 2012; Popov et al., 2015). Most water molecules directly bind to glycerol via hydrogen bonding, but excess water, which is phase separated from glycerol-water mixtures, would be ascribed to the cold-crystallization. It is also known that this kind of water is so-called intermediate water (Tanaka et al., 2013, 2015). This crystallization is a kinetic (not equilibrium) phenomenon. So, the observation is dependent on cooling and heating rates. The intensity of the HOH-bending peak increases linearly as a function of Aw in region a, whereas a deviation from linearity is observed in region b. This might imply the percolation of hydrogen bonding in water molecules, because the percolating water exhibits highly mobility compared with the isolated water or small clusters of water (Nakagawa and Kataoka, 2010). In region c (Aw > ≈0.8), water is essentially bulk water; the FWHM of water molecules in the IQENS analysis is significantly larger than that in regions a and b. This likely indicates that the water molecules have faster dynamics like in bulk water. The dynamical change of water molecules is likely coupled with aggregated water domains (Jensen et al., 2018). Furthermore, a negligible EISF means that water molecules are not restricted in confined space at the pico-second time scale. These results indicate that in the region c, water molecules would travel with larger distance in the pico-second time scale in the solution due to the weaker hydrogen bonding between glycerol and water and/or water molecules. This dynamical change observed by IQENS is correlated with the localized dynamical change observed in ATR-IR. The weak interaction of water with glycerol and the water lead to fewer dynamic constraints of water at the molecular scale and consequently lead to higher Aw. In food systems, at the higher Aw, the thermal fluctuation of water is more significant, and the forming and breaking of the hydrogen bonding with food ingredients is facilitated. These dynamical interactions might degrade the quality of food. The mobile water should plasticize the food in the amorphous state (Roos, 1993). This effect would lead to the degradation of the food due to the faster relaxation via hydrogen bonding. It should be essential to discuss the structure-dynamics-quality of food from the microscopic view in the basic food science. The present work should provide the new view about the interpretation for water activity, which should be a significant breakthrough. It should be noted that the FWHM in IQENS follows a similar behavior to that of the sorption isotherm. IQENS is an effective method for elucidating the physical basis of Aw, enabling the analysis of water dynamics in food systems.

## Conclusions

The physical properties of water in glycerol–water mixtures are examined, and three regions are classified in the sorption isotherm. Values of Aw are correlated with the thermodynamics and molecular dynamics of water molecules in the solutions. Water clustering and network formation (percolation of hydrogen bonding in water molecules) as well as the interaction of water with glycerol determine the Aw. It should be noted that IQENS provided fruitful dynamical information in time and space scales to explain the molecular basis of Aw in the solutions. IQENS is a promising method in food science.

## Data Availability Statement

All datasets generated for this study are included in the article/supplementary material.

## Author Contributions

HN designed the research, performed water activity measurement, DSC and neutron scattering experiment. TO performed ATR-IR measurement. HN and TO discussed the experimental data together. HN wrote the first manuscript. HN and TO improved the manuscript together.

### Conflict of Interest

TO was employed by company Jasco Corporation. The remaining author declares that the research was conducted in the absence of any commercial or financial relationships that could be construed as a potential conflict of interest.
